# Prognostic Value of Conventional Ultrasound and MRI Features for Clinical Outcomes in Athletes With Patellar Tendinopathy After Exercise Therapy

**DOI:** 10.1177/19417381251401164

**Published:** 2026-01-26

**Authors:** Jie Deng, Stephan J. Breda, Yijie Fang, Denise Eygendaal, Robert-Jan de Vos, Edwin H.G. Oei

**Affiliations:** † Department of Radiology and Nuclear Medicine, Erasmus MC University Medical Center, Rotterdam, the Netherlands; ‡ Department of Orthopedics and Sports Medicine, Erasmus MC University Medical Center, Rotterdam, the Netherlands; § Department of Radiology, AZ Turnhout, Belgium; ‖ Department of Radiology, the Fifth Affiliated Hospital, Sun Yat-sen University, Zhuhai, Guangdong, China

**Keywords:** clinical course, imaging parameters, knee pain, recovery

## Abstract

**Background:**

Structural abnormalities assessed with conventional ultrasound (US) or magnetic resonance imaging (MRI) are associated with the risk of developing patellar tendinopathy (PT). However, their prognostic value for athletes with PT performing exercise therapy remains unclear.

**Hypothesis:**

Baseline imaging features could be associated with changes in pain and disability over 24 weeks in athletes with PT after exercise treatment.

**Study Design:**

Cohort study.

**Level of Evidence:**

Level II.

**Methods:**

Athletes with PT were randomly allocated to 2 different programs of exercise therapy for 24 weeks. Imaging features at baseline included patellar tendon thickness, intratendinous calcifications, patellar erosions, and Doppler flow on US, as well as tendon fiber disruption, infrapatellar fat pad (IFP) edema, bone marrow edema, and deep infrapatellar bursitis on MRI scan. Clinical outcomes were measured at baseline, and at 12- and 24-week follow-up, using the visual analog scale after single-leg squat (VAS-SLDS) for pain on loading, and Victorian Institute of Sports Assessment-Patella (VISA-P) questionnaire for disability. Linear mixed-effects models, incorporating interaction terms tested using likelihood ratio tests, evaluated the prognostic value of baseline imaging features.

**Results:**

Of 76 included athletes (58 male, 18 female; average age, 24 ± 4 years), abnormal US features were identified in 26% to 78% of cases. Among 72 MRI scans analyzed, abnormal features were demonstrated in 43% to 81% of cases. No significant associations were identified between individual imaging features and 24-week changes in VAS-SLDS or VISA-P scores (all *P*_interaction_ > 0.10), or between the total number of imaging abnormalities and clinical outcomes (all *P*_interaction_ > 0.50).

**Conclusion:**

There was no evidence of an association between baseline abnormalities assessed using conventional US or MRI and 24-week changes in pain or disability among athletes with PT undergoing exercise therapy.

**Clinical Relevance:**

Healthcare professionals should avoid relying on conventional imaging findings to predict prognosis.

Patellar tendinopathy (PT) is a highly prevalent injury in jumping athletes.^32^ This disease is characterized by long-lasting pain and disability and typically has a negative impact on sports performance and work participation.^19^ Exercise therapy is considered the first-line treatment in PT.^12,18,30^ The clinical course of pain and disability in PT athletes after this standard treatment is often characterized by modest improvement in average pain and disability at 3 months of follow-up,^11^ with little or no further change during long term.^1^ Substantial inter-patient variations exist in these outcomes, suggesting that some athletes experience meaningful recovery while others show minimal response.^1,16,30^ This variability highlights the need for prognostic research to identify factors that can help distinguish between individuals with a favorable and unfavorable prognosis.^35^ This would facilitate patient education and optimize treatment recommendations.

Patients with PT commonly demonstrate structural changes or increased vascularization in the patellar tendon. Previous studies have found that tendon thickening and neovascularization on ultrasound (US) are associated with a higher risk of developing PT.^14,29^ In addition, US-detected intratendinous calcification or patella erosions in PT patients may indicate the level of disease chronicity.^9^ Magnetic resonance imaging (MRI) is also used in PT to identify pathological changes in adjacent structures, such as infrapatellar fat pad (IFP, or Hoffa’s fat pad) edema,^13^ and bone marrow edema.^33^ Besides, the ratio of partial tear thickness to overall tendon thickness on MRI scans (ie, >50% tear thickness) may serve as a classifier for surgical decision-making in athletes with PT.^22^ Although these features are commonly used to assist in clinical diagnosis, their prognostic value for predicting clinical outcomes after exercise treatment in PT athletes remains poorly understood.

Therefore, this study aimed to examine the prognostic value of structural abnormalities detected by conventional US and MRI at baseline for the 24-week change in pain and disability in athletes with PT following exercise treatment.

## Methods

### Study Design and Participants

This is a secondary analysis of a randomized controlled trial of 76 athletes with PT performing progressive tendon-loading exercises (PTLE) or eccentric exercise therapy (EET) at Erasmus MC University Medical Center between January 2017 and July 2019 (ClinicalTrial.gov: NCT02938143).^7^ The PTLE group consisted of isometric loading, isotonic loading, energy-storage loading, and sport-specific exercises. Athletes performing the EET program were advised to perform a single-leg squat using a 25° decline board and sport-specific exercises. Both groups performed their prescribed exercises for 24 weeks in an unsupervised setting. However, based on their own preference, they arranged supervision by a health professional. Details of the exercise treatment strategies were previously published.^7^ All participants provided informed consent for the study, which was approved by the institutional review board (MEC-2016-500). For this secondary analysis, we combined patients undergoing both exercise treatments into 1 cohort to enhance statistical power and maximize the available data. Given that the intervention group demonstrated statistically superior clinical improvement at 24 weeks,^7^ we accounted for potential differences by including treatment allocation as a covariate in the model.^31^ We adhered to the minimum reporting standards for tendinopathy trials according to the international consensus statement.^37^ This study was performed and reported in accordance with the guidelines for assessing prognostic factors under the PROGRESS framework.^35,36^

Consecutive athletes aged 18 to 35 years with PT (characterized by pain on loading and palpation pain on the inferior part of the patella, the Victorian Institute of Sports Assessment-Patella (VISA-P) questionnaire <80 points) confirmed by US (tendon structural changes and/or anterior-posterior thickness >6 mm in grayscale US and/or increased vascularity by power Doppler) were enrolled. Main exclusion criteria included coexisting knee pathologies on US or MRI (eg, cartilage lesions, full-thickness patellar tendon rupture), as these could confound the diagnosis or outcome of patellar tendinopathy. We also excluded participants with a history of joint injection therapy or ≥4 weeks of structured exercise therapy in the previous 12 months, to minimize residual effects from previous treatments. Additional exclusion criteria: previous knee surgery without full rehabilitation, previous patellar tendon rupture, acute knee or patellar tendon injury, or inability to participate in an exercise program, either due to safety concerns or to ensure adherence to the intervention protocol.

### Measurements

#### Clinical Outcome Measures

Pain and disability related to PT were measured at baseline, 12 weeks, and 24 weeks using pain intensity rated on the Visual Analog Scale directly after the single-leg decline squat (SLDS) (VAS-SLDS) and VISA-P score, respectively. These outcome measures align with a reported consensus for core domains for tendinopathy.^44^ Patients performed the SLDS test when they completed the VISA-P questionnaire before the imaging examination was done, so they were not aware of the imaging findings when they reported the clinical severity of their symptoms at that specific timepoint.

#### Ultrasound

**Figure 1.**
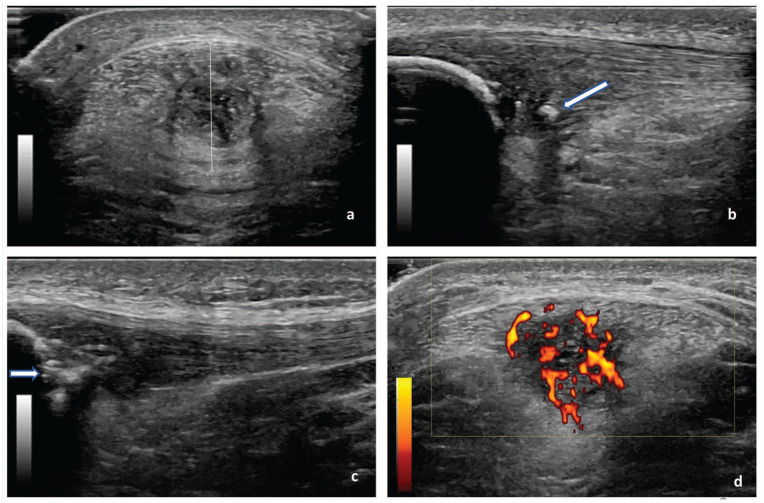
Patellar tendon assessed by US: (a) AP thickness measured in the transverse plane, (b) intratendinous calcifications (arrow) identified in the sagittal plane, and (c) patella erosions (arrow) identified in the sagittal plane. (d) PDUS: high level of Doppler flow (modified Ohberg score, 4) assessed in the transverse plane. These images are from different athletes with PT. AP, anteroposterior; PDUS, power Doppler US; PT, patellar tendinopathy; US, ultrasound.

Grey-scale US (GSUS) and power Doppler US (PDUS) were performed at baseline and conducted in the longitudinal and transverse planes using a linear 5 to 15 MHz transducer (GE Healthcare, ML6-15; LOGIQ E9), with a US gel (Sonogel Vertriebs GmbH).^6^ All participants were in the supine position with the back rest of the examination table upright at 60° for patient comfort. Both knees were placed in 30° flexion to limit anisotropy, supported by a foam roll. The imaging was acquired at 15 MHz, with a fixed depth of 2.5 cm, a gain of 41 dB, auto-optimization set to 100%, and a dynamic range of 66 dB. Patellar tendon and surrounding tissues were evaluated on US as follows^9,20^: (1) anteroposterior (AP) thickness of patellar tendon was measured in the transverse plane at the thickest point within 1 cm distal to the inferior pole of the patella; (2) intratendinous calcifications, hyperechoic in the patellar tendon with posterior acoustic shadowing; and 3) patella erosions, a cortical discontinuity or fragmentation of the inferior pole of the patella in the longitudinal or transverse plane (Figure 1a-c).

PDUS was acquired in the transverse plane at 10 MHz, with 18 dB gain, a pulse repetition frequency of 1.0 kHz, and wall filters at 128 Hz. The box size of the region of interest (ROI) was 3.4 cm wide and 1.8 cm deep (Figure 1d). Participants were asked to extend both knees to increase the sensitivity of detecting (peri) tendinous blood flow.^40^ The modified Ohberg score was used to rate the tendinous Doppler signal using 5 scores (Table S1, Appendix A, available in the online version of this article).^46^

#### Magnetic Resonance Imaging

MRI was performed at baseline using a 3.0-T scanner (Discovery MR750, GE Healthcare). All MRI scans were performed according to a previously described MRI protocol.^8^ The protocol included the following pulse sequences: three-dimensional (3-D) fat-suppressed proton density-weighted sequence and axial fat-suppressed T2-weighted sequence (parameters for these 2 sequences are described in Table S2, Appendix A, available online).

**Figure 2.**
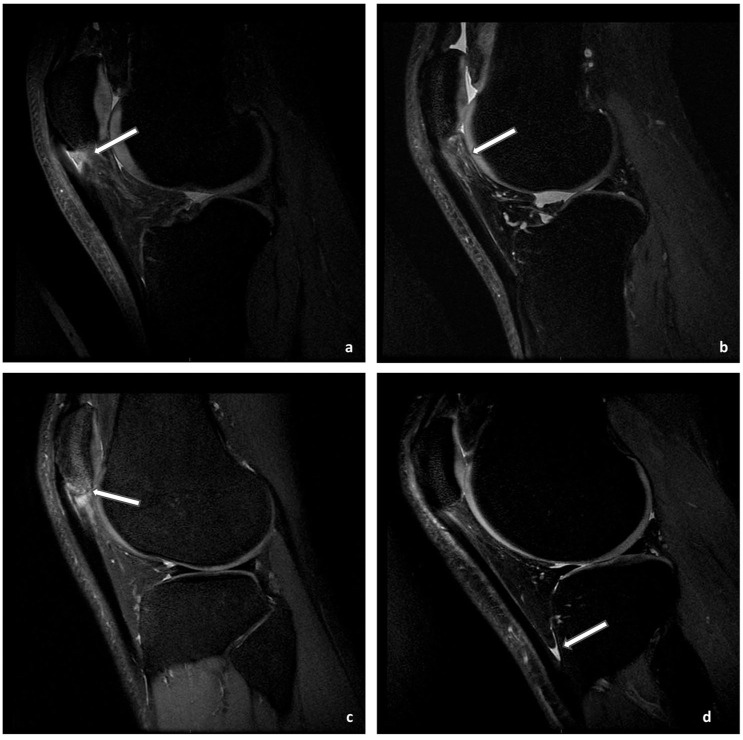
Sagittal, fat-suppressed proton density MRI scans: (a) focal tendon disruption (arrow), (b) grade 2 IFP (or Hoffa fat pad) edema (arrow), (c) the presence of bone marrow edema (arrow), and (d) the presence of deep infrapatellar bursitis (arrow). These images were from different athletes with PT. IFP, infrapatellar fat pad; MRI, magnetic resonance imaging; PT, patellar tendinopathy; US, ultrasound.

The patellar tendon and surrounding tissues were evaluated for the following features: (1) focal tendon disruption: loss of fiber continuity on axial or sagittal images. In addition, >50% focal tendon disruption was also collected in the axial view (1 slice below the inferior pole of the patella) according to the previous literature^22^; (2) IFP edema: any area of increased signal intensity in the Hoffa fat pad, using a 4-point graded scale (Table S1, Appendix A, available online)^15^; (3) bone marrow edema: increased signal intensity in the bone marrow of the inferior pole of the patella^34^; and (4) deep infrapatellar bursitis: increased signal in the distended bursa (Figure 2).^2^

### Imaging Analyses

All US and MRI scans were reviewed on our institutional picture archiving and communication system (PACS). An electronic caliper tool in PACS was used to determine AP patellar tendon thickness. All US images were analyzed by 1 musculoskeletal radiologist (S.J.B.) with 5 years of experience and 1 sports physician (J.D.) with 3 years of experience. One musculoskeletal radiologist (Y.F.) with 11 years of experience and the same sports physician analyzed all MRI scans. Disagreements were mutually debated and solved with a third musculoskeletal radiologist (E.H.G.O.) with 20 years of experience. All these evaluators were blinded to the clinical outcomes at the time of image analysis.

### Statistical Analyses

Descriptive statistics for continuous data were presented as means and standard deviations for normally distributed data evaluated by visual check using histograms and also assessed by the Shapiro-Wilk test. Otherwise, medians and interquartile ranges were used.

We used linear mixed-effects models to evaluate the trajectories of VAS-SLDS and VISA-P scores over 24 weeks. The basic model included time and time-dependent covariates, including treatment allocation, sex, age, body mass index (BMI), symptom duration, and sports activity as fixed effects.^3^ The baseline value of outcome and each imaging feature were added to the basic model to assess the prognostic value of imaging factors. The difference in the 24-week change of outcomes between participants with and without imaging abnormalities was assessed by including an interaction term (time × imaging factor) into the model. The significance of this interaction was then tested using a likelihood ratio test (LRT), comparing models with and without the interaction term. All models included random intercepts and random slopes of time (constant up to 24 weeks) at the individual level. For AP thickness as a continuous predictor, we modeled nonlinearity by fractional polynomials.^45^ To facilitate clinical interpretation and enhance statistical power given sparsity in some ordinal categories, we dichotomized 3 predictors: AP thickness (diameter >6 mm) for tendon thickening,^20^ Doppler flow (no to low [score 0 to 2] vs modest to high [score 3 to 4]),^17^ and IFP edema (no to low [grade 0 to 1] vs modest to high [grade 2-3]). Regression analysis was not performed for >50% focal tendon disruption due to its low prevalence (n = 3; 4%). We had also planned to assess the association between the presence of a hypoechoic region on US and increased signal intensity on MRI at the patellar tendon (ie, features consistent with tendinopathy). However, all included athletes demonstrated these findings at baseline as it was an inclusion criterion, resulting in no variability in our study population. As a result, these features could not be evaluated as predictors in the regression models. We further evaluated the association between the number of imaging abnormalities (none, 1-2 abnormalities, 3-4 abnormalities, and ≥5 abnormalities) and the change in outcomes.

Linear mixed-effects models can handle missing outcomes when the mechanism of missingness is missing at random. However, missing values in MRI features at baseline (see Table 1) were imputed using multiple imputation. For the imputation model, we included the abovementioned covariates and auxiliary variables (details in Appendix A, available online) and generated 20 imputed datasets. The results from each imputed dataset were pooled by Rubin’s rules.^41^ We repeated the primary analyses using complete cases as sensitivity analyses. The inter-rater reliability (IRR) for imaging findings among 2 raters was evaluated using Cohen’s kappa for binary variables, Gwet’s AC2 for ordinal outcomes,^5^ and interclass correlation coefficient (ICC) for continuous variables.^4^ These values range from 0 to 1, and the responding cut-off values are described in Table S3, Appendix A, available online.

**Table 1.** Baseline demographic, clinical, and imaging features in the study cohort (n = 76)

	Values
Demographics	
Age, years	24 ± 4
Male, n (%)	58 (76)
BMI, kg/m^2^	24 ± 3
Clinical factors	
Symptom duration, weeks	104 [49-208]
Sports activity level by CSAS, n (%)	
Level 1: 4-7 days per week	17 (22)
Level 2: 1-3 days per week	59 (78)
VAS-SLDS, (0-10)	5 ± 2
VISA-P score, (0-100)	55 ± 13
US	
AP thickness, mm	9 ± 2
Thickening >6 mm, n (%)	59 (78)
The presence of intratendinous calcification, n (%)	20 (26)
The presence of patella erosions, n (%)	24 (32)
Power Doppler flow, n (%)	
Level: no to low	25 (33)
Score 0	6 (8)
Score 1	2 (3)
Score 2	17 (22)
Level: moderate to high	51 (67)
Score 3	7 (9)
Score 4	44 (58)
MRI*^a^*, n (%)	
Presence of focal tendon disruption	58 (81)
>50% focal tendon disruption	3 (4)
IFP edema	
Level: no to low	23 (32)
Grade 0	8 (11)
Grade 1	15 (21)
Level: moderate to high	49 (68)
Grade 2	19 (26)
Grade 3	30 (42)
The presence of bone marrow edema	31 (43)
The presence of deep infrapatellar bursitis	37 (51)

Values presented as mean ± SD, n (%), or median [IQR]. AP, anteroposterior; CSAS, Cincinnati Sports Activity Scale; IFP, infrapatellar fat pad; MRI, magnetic resonance imaging; US, ultrasound; VAS-SLDS, Visual Analogue Score after single-leg decline squat; VISA-P, Victorian Institute of Sports Assessment-Patella.

a Total sample size for athletes with MRI is 72.

We did not apply multiple testing correction due to the fact that these were exploratory analyses. Linear mixed-effects models were diagnosed by residual plots. All statistical analyses were conducted in R Studio Version 2023.12.0 with ‘mice,’ ‘nlme,’ ‘mitml,’ ‘irr,’ and ‘irrCAC’ packages. A *P* value of <0.05 suggested statistical significance.

## Results

### Baseline Characteristics and Findings of Imaging

**Figure 3.**
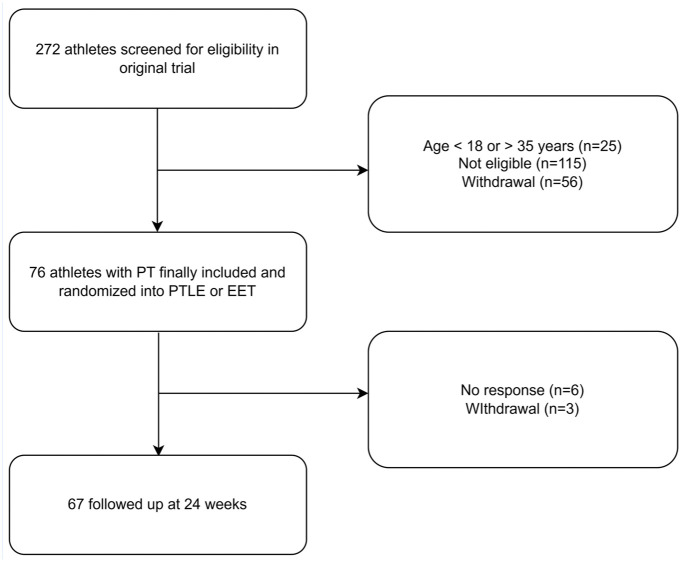
Flow chart of the study. EET, eccentric exercise therapy; PT, patellar tendinopathy; PTLE, progressive tendon loading exercises.

A total of 76 PT athletes were studied (Table 1). Figure 3 shows the study flowchart. The response rates at 12 and 24 weeks were 84% and 88%, respectively.

Among the 76 US examinations analyzed (100%), abnormal US features were identified in 26% to 78% of cases (Table 1). For MRI assessment, 72 out of 76 scans were available (95%, 4 were missing due to scanner unavailability caused by urgent clinical cases and time constraints). Among the 72 MRI scans analyzed, abnormal MRI features were demonstrated in 43% to 81% of athletes. All imaging features demonstrated good IRR (Table S1, Appendix B, available online).

### Association Between any Imaging Feature and 24-week Change in Pain and Disability

The estimated mean change from baseline to 24 weeks was a decrease of 3 points (95% CI, 2 to 3; *P* < 0.001) in VAS-SLDS and an increase of 23 points (19 to 27; *P* < 0.001) in VISA-P score.

**Figure 4.**
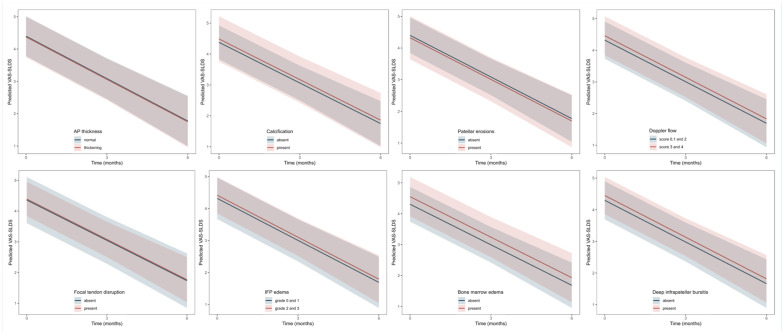
Effect plots visualize the 24-week decrease in VAS-SLDS according to the presence or absence of imaging features. Parallel lines suggested that the extent of change in VAS-SLDS was similar between groups, indicating no prognostic value of any of these imaging features. The 95% CIs are calculated from linear mixed-effect models using shaded areas. AP, anteroposterior; IFP, infrapatellar fat pad; VAS-SLDS, Visual Analog Score after single-leg decline squat.

None of the evaluated imaging features was associated significantly with VAS-SLDS at 12 or 24 weeks (β values range: –0.1 to 0.2; all *P* values > 0.10; Table 2). Moreover, interaction terms were not statistically significant (all *P*_interaction_ > 0.10), suggesting the presence of these imaging abnormalities at baseline did not significantly influence the 24-week change in VAS-SLDS compared with those without such features (Figure 4, Table S2, Appendix B, available online).

**Table 2.** Results of linear mixed-effect models with 24-week change in clinical outcomes according to imaging features at baseline

Imaging features	VAS-SLDS			VISA-P score		
	β (95% CI)*^a^*	*P* value	*P* value for interaction*^b^*	β (95% CI)*^a^*	*P* value	*P* value for interaction*^c^*
AP thickness, continuous	NA	NA	0.72	NA	NA	0.21
AP thickness, categorical			0.91			0.40
Tendon thickening vs normal	–0.0 (–0.61 to 0.56)	0.93		–1.3 (–4.64 to 2.08)	0.46	
Intratendinous calcifications			0.85			0.13
Present vs absent	0.1 (–0.51 to 0.63)	0.78		–0.2 (–3.41 to 2.95)	0.89	
Patellar erosions			0.16			0.28
Present vs absent	–0.1 (–0.60 to 0.44)	0.68		1.1 (–1.91 to 4.21)	0.47	
Power Doppler flow			0.31			0.35
Moderate to high vs no to low	0.1 (–0.37 to 0.65)	0.60		1.2 (–1.81 to 4.21)	0.44	
Focal tendon disruption			0.11			0.99
Present vs absent	0.0 (–0.58 to 0.64)	0.91		–1.3 (–4.65 to 2.11)	0.46	
IFP edema			0.44			0.12
Moderate to high vs no to low	0.1 (–0.39 to 0.59)	0.69		–0.5 (–3.46 to 2.48)	0.75	
Bone marrow edema			0.37			0.94
Present vs absent	0.2 (–0.22 to 0.70)	0.30		–1.2 (–3.94 to 1.54)	0.39	
Deep infrapatellar bursitis			0.15			0.51
Present vs absent	0.2 (–0.31 to 0.61)	0.52		–0.0 (–2.82 to 2.80)	0.10	

AP, anteroposterior; IFP, infrapatellar fat pad; NA, not applicable; ; US, ultrasound VAS-SLDS, Visual Analog Score after single-leg decline squat; VISA-P, Victorian Institute of Sports Assessment-Patella.

a The coefficients are derived from a linear mixed-effect model without an interaction term (imaging factor × time), representing the association between imaging factor and outcomes during follow-up (12 or 24 weeks).

b Likelihood ratio test for VAS-SLDS, comparing models with and without the interaction term (imaging factor × time), indicating the difference in 24-week change of VAS-SLDS between people with and without imaging factors.

c Likelihood ratio test for VISA-P score, comparing models with and without the interaction term (imaging factor × time), indicating the difference in 24-week change of VISA-P score between people with and without imaging factors.

**Figure 5.**
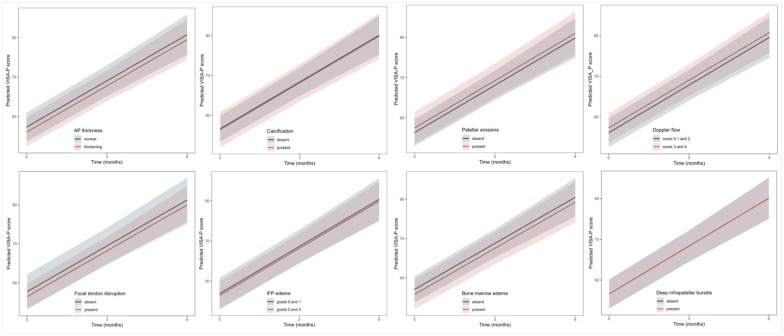
Effect plots visualize the 24-week increase in VISA-P score according to the presence or absence of imaging features. Parallel lines suggested that the extent of change in VISA-P score was similar between groups, indicating no prognostic value of any of these imaging features. The 95% CIs are calculated from linear mixed-effect models using shaded areas. AP, anteroposterior; IFP, infrapatellar fat pad; VISA-P, Victorian Institute of Sports Assessment-Patella.

Similar to VAS-SLDS, these imaging features were not associated significantly with VISA-P scores at 12 or 24 weeks (β values range: –1.3 to 1.2; all *P* values > 0.05; Table 2), nor did they modify the course of in VISA-P scores over 24 weeks (all *P*_interaction_ > 0.10; Figure 5, Table S2, Appendix B, available online).

Sensitivity analyses yielded similar results, although the precision of the estimates in cases with complete MRI data was generally lower (Table S3, Appendix B, available online).

### Association Between the Number of Abnormal Imaging Features and 24-Week Change in Pain and Disability

**Figure 6.**
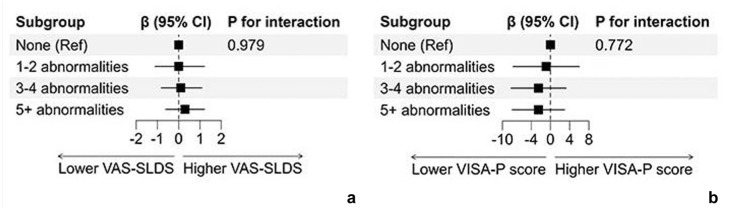
Forest plots visualize the association between the number of imaging abnormalities and outcomes during 24 weeks. No significant interaction term indicates that the 24-week change in (a) VAS-SLDS or (b) VISA-P score did not differ according to the number of imaging abnormalities. VAS-SLDS, Visual Analog Score after single-leg decline squat; VISA-P, Victorian Institute of Sports Assessment-Patella.

When combining features from US and MRI, no abnormalities were observed in 5 out of 72 participants (7%). Among those with abnormalities (n = 67), 6 (9%) had 1 or 2 features, 23 (34%) had 3 or 4 features, and 38 (57%) had ≥5 features. PT athletes with a coexistence of multiple imaging abnormalities tended to report generally higher pain levels and greater disability during the follow-up timepoint. However, these differences were not statistically significant (Figure 6). The 24-week change in VAS-SLDS or VISA-P scores did not differ significantly according to the number of imaging abnormalities (all *P*_interaction_ > 0.50; Figure 6).

## Discussion

To our knowledge, this is the first study investigating the prognostic value of conventional US or MRI findings for predicting the 24-week change of pain and disability in PT athletes undergoing exercise treatment. Our findings revealed that, despite the high prevalence (26-81%) of abnormal imaging features in our study population, no single imaging feature was significantly associated with longitudinal change in clinical outcomes. Specifically, neither pain reduction measured by VAS-SLDS nor disability improvement assessed by VISA-P scores differed significantly between athletes with and without abnormal imaging features. In addition, the number of abnormal imaging features did not significantly influence the 24-week changes in pain and disability. These results highlight that features identified by conventional imaging modalities may have limited utility in predicting recovery after exercise treatment for PT, despite the fact that many selected features are risk factors for developing PT.^14,24,29^ These findings are crucial for optimizing patient education in the clinical setting.

### The Prognostic Value of Structural Change in the Patellar Tendon

Previous research investigating the potential prognostic value of structural abnormalities in PT has been limited to tendon thickening and partial tendon tears.^22^ This is not surprising because an enlarged tendon is one of the structural changes observed most commonly in PT patients,^38^ and increased tendon thickness may correlate with the presence of tears.^22^ To the best of our knowledge, only 1 previous study in PT has explored the relationship between these structural abnormalities and return to activity or sport after nonoperative treatment. Golman et al^22^ reported that tendon thickness (>11.5 mm) or substantial partial tear (>50% tear thickness) on MRI was a significant classifier for surgical treatment for PT patients. However, this retrospective and lack of longitudinal design limits the ability to establish these imaging features as prognostic factors,^23^ as clinical decisions to pursue surgery may be influenced by clinician judgment, and the observed associations could be confounded by unmeasured variables. We did not find tendon thickness or the presence of a focal tear to be associated with a worse prognosis. Our findings are interpreted more reliably as prognostic, as we adhered to current guidelines for assessing prognostic factors,^23,35,36^ and accounted for potential nonlinear relationships between tendon thickness and clinical outcomes in PT,^39,45^ which could induce erroneous inference.^25^ While it is worthwhile mentioning that, compared with the study of Golman et al^22^, our sample included few sufficient representations of severe cases (the mean tendon thickness was 8.8 mm [SD, 2.3], and only 3 cases [4%] had >50% tear thickness). All 3 cases improved with exercise therapy, and none needed surgery (Figure S1, Appendix B, available online). Although conclusions cannot be drawn due to the very small sample size, these descriptive findings illustrate that partial tendon ruptures do not necessarily require surgical intervention.

### The Prognostic Value of Neovascularization

Although no previous research has directly evaluated the prognostic significance of neovascularization in PT patients performing exercise therapy, several studies have focused primarily on the effect of treatment targeting the eradication of neovascularization. A small-scale randomized trial found borderline statistical significance for improved VISA-P scores at 4 months in PT patients receiving sclerosing injections aimed at reducing neovascularization compared with those receiving sham injections.^26^ Two prospective cohort studies reported improvements in pain and disability scores following sclerotherapy or electrocoagulation for neovascularization.^10,27^ Taken together, these findings suggest that interventions that reduce neovascularization could improve clinical outcomes, thereby indirectly supporting a prognostic role. However, our results did not confirm the prognostic value of neovascularization assessed with PDUS. One possible explanation for this discrepancy could be that 2 out of these 3 previous studies lacked control groups,^10,27^ making it difficult to determine whether the observed improvements were due to natural history or a true therapeutic effect from reducing neovascularization. Another reason might be our use of a dichotomized classification, categorizing neovascularization into “no to low” (scores 0 to 2) and “moderate to high” (scores 3 to 4). This simplified grouping may have limited our ability to discern the specific prognostic relevance of the presence versus absence of neovascularization. However, this methodological decision was aligned with our previous research and was necessary to improve statistical model stability in our case,^17^ with only 8% of cases demonstrating no detectable Doppler signal and potential false positives at low-grade neovascularization level (score 1, 3%).^21^ Future large-scale studies employing a more refined categorization of neovascularization might better clarify its specific prognostic impact on rehabilitation outcomes in PT.

### The Prognostic Value of Abnormalities in Adjacent Structures

No studies have examined the association between MRI-assessed abnormalities in adjacent structures and clinical outcomes in athletes with PT following exercise treatment. Only 1 prospective cohort (n = 30) found that preoperative IFP edema, or the coexistence of IFP and bone marrow edema in PT patients, was associated with worse pain and disability at a mean follow-up of 4 years after surgery.^33^ Although it is debatable whether prognostic factors from the study population under different treatments can be compared directly, the previous study did not employ regression analyses, preventing its prognostic validity.^36^ Considering the latter study included primarily chronic or refractory cases who had failed ≥6 months of nonoperative treatment,^33^ it remains uncertain whether the reported associations reflect a true or independent prognostic relationship or are distorted by the underlying confounders such as chronicity of symptoms,^43^ greater functional impairments, and possible concurrent psychological or social factors that may predict prognosis in tendinopathy.^28^

### Strengths and Limitations

One of the key strengths of our study is that it was performed in accordance with the recommendations for assessing prognostic factors under the PROGRESS framework.^35,36^ By employing a prospective design with longitudinal outcomes, adjusting for confounders, and using imputation methods to address missing prognostic factors, we can reliably evaluate prognostic factors. Another strength is that we predefined 2 clinical outcomes that align with the core domains of tendinopathy.^44^ The main limitation is that imaging features were assessed using binary classifications (presence or absence) rather than (semi-)quantitative measures, such as the area of vascular signal.^42^ Employing quantitative assessments might have provided a more in-depth understanding of the prognostic significance of these imaging features. In addition, although the imaging features showed no prognostic value, the absence of a control group (eg, patients receiving nonexercise treatment) limits our ability to assess whether these features modify the effect of exercise treatment (treatment effect modifier). Finally, due to a lack of established grading systems for classifying imaging abnormalities into specific subgroups, our categorization of coexisting imaging abnormalities into 4 levels (none, 1-2 abnormalities, 3-4 abnormalities, and ≥5 abnormalities) was data driven. Therefore, the clinical relevance of these subgroupings should be interpreted with caution, and validation in future studies is warranted.

### Clinical Implications and Future Research

Despite the high prevalence of structural and vascular abnormalities on US and MRI scans in athletes with PT, these imaging features were not associated with the degree of clinical improvement after exercise therapy. Clinicians should be aware that these imaging findings do not reliably predict individual treatment outcomes. Patients should therefore be informed that the presence of these conventional imaging abnormalities has a limited ability to determine their likelihood of recovery. This may help set realistic expectations and avoid unnecessary concern. Future research should include comparison groups receiving nonexercise-based interventions to assess whether imaging features act as treatment effect modifiers, and explore whether more refined or quantitative imaging metrics could better identify subgroups who respond differently to specific treatment approaches.

## Conclusion

In this large prospective cohort study, we found no evidence that abnormalities assessed using conventional US or MRI were associated with 24-week changes in pain or disability among athletes with patellar tendinopathy undergoing exercise therapy. Although a substantial proportion of participants exhibited multiple coexisting imaging abnormalities, the number of abnormalities did not influence clinical outcomes over time. These findings indicate that commonly used imaging features in clinical practice lack prognostic value in this context. Healthcare professionals should avoid relying on these conventional imaging findings to predict prognosis. Patients can be informed that, despite the presence of structural or vascular abnormalities on imaging, they could still have a good chance of meaningful recovery with exercise-based treatment.

## Supplemental Material

sj-docx-1-sph-10.1177_19417381251401164 – Supplemental material for Prognostic Value of Conventional Ultrasound and MRI Features for Clinical Outcomes in Athletes With Patellar Tendinopathy After Exercise Therapy Supplemental material, sj-docx-1-sph-10.1177_19417381251401164 for Prognostic Value of Conventional Ultrasound and MRI Features for Clinical Outcomes in Athletes With Patellar Tendinopathy After Exercise Therapy by Jie Deng, Stephan J. Breda, Yijie Fang, Denise Eygendaal, Robert-Jan de Vos and Edwin H.G. Oei in Sports Health
