# Supplementary material for: Prognostic Value of Conventional Ultrasound and MRI Features for Clinical Outcomes in Athletes With Patellar Tendinopathy After Exercise Therapy
Source: Sports Health. 2026 Jan 26:19417381251401164. Online ahead of print. doi: 10.1177/19417381251401164 (PMC12846899; doi:10.1177/19417381251401164)
Supplement: sj-docx-1-sph-10.1177_19417381251401164 – Supplemental material for Prognostic Value of Conventional Ultrasound and MRI Features for Clinical Outcomes in Athletes With Patellar Tendinopathy After Exercise Therapy [file sj-docx-1-sph-10.1177_19417381251401164.docx]

**Supplementary file**

**Appendix A: methods**

**Multiple imputation**

We performed multiple inputation using chained equations for missing MRI data. For the imputation model, we included confounders described in the method in the main text and auxiliary variables (sports participation in desired sport at baseline, sonographic imaging factors: tendont thickness, intratendinous calcification, patellar erosions and Doppler flow). The results from each imputed dataset were pooled based on Rubin’s rule. For the likelihood ratio test, we used *D*1, considering we have a small sample size [1].
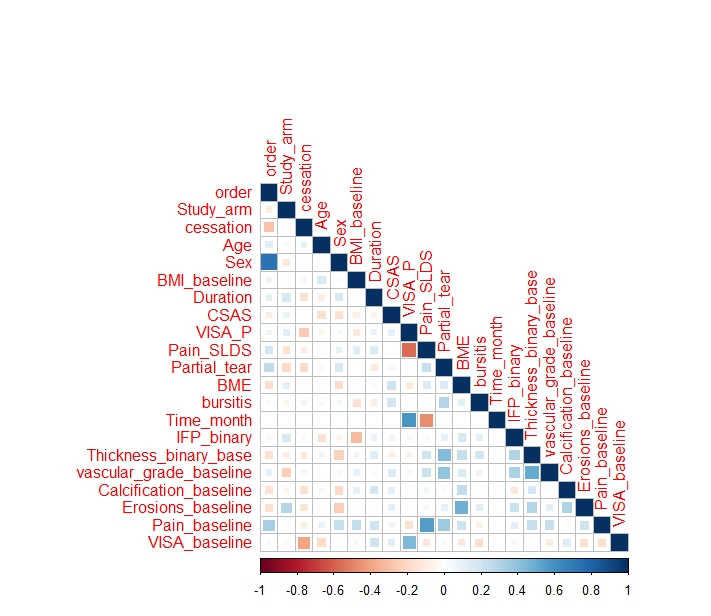


**Figure S1: Correlation plot among variables in the imputation model**


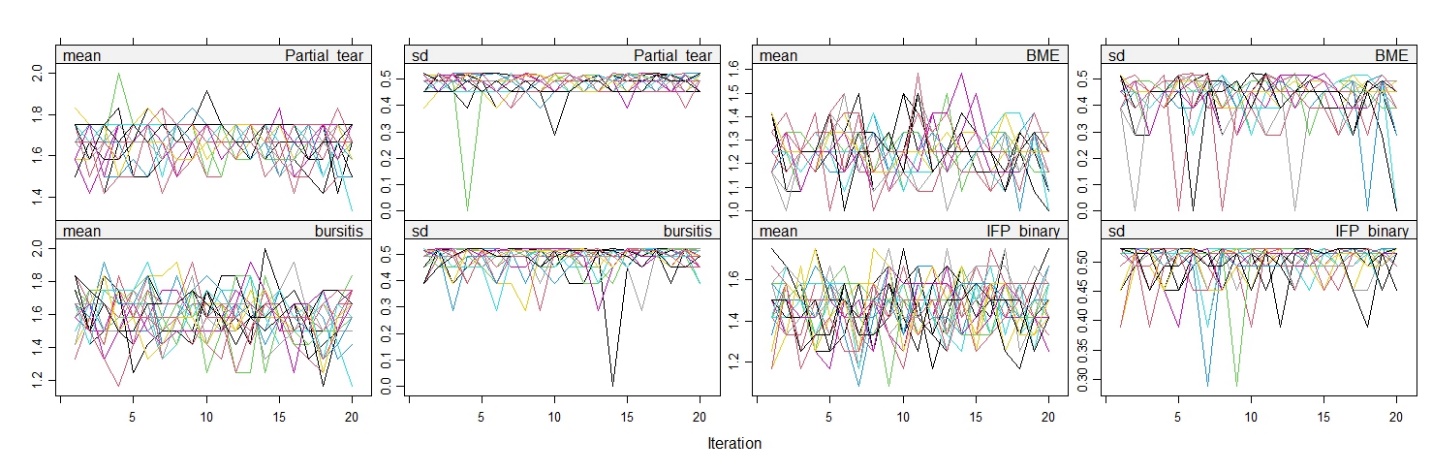


**Figure S2. Convergence plot of 20 imputed dataset**

[1] van Buuren, S. (2018). Flexible Imputation of Missing Data, Second Edition (2nd ed.). Chapman and Hall/CRC. <https://doi.org/10.1201/9780429492259>

**Table S1. Grey-scale ultrasound, power Doppler imaging, and MRI grading scheme**

| **Imaging features** | **Measuring units or grading scores** |
| --- | --- |
| **Ultrasound** | |
| Tendon AP thickness, continuous  Tendon AP thickness, categorical | Normal (diameter ≤ 6 mm)  Tendon thickening (diameter > 6 mm) |
| Intratendinous calcification(s) | Absent  Present |
| Patella erosions | Absent  Present |
| Doppler flow | No to low  Score 0: no vessels  Score 1+: one vessel posterior to the patella tendon  Score 2+: one to two vessels throughout the tendon  Moderate to high  Score 3+: three vessels throughout the tendon  Score 4+: four or more vessels throughout the tendon |
| **MRI** | |
| Focal tendon disruption  >50% focal tendon disruption | Absent  Present  Absent  Present |
| IFP edema | No to low  Grade 0: no edema  Grade 1: subtle increased signal intensity  Moderate to high  Grade 2: definite increased signal intensity  Grade 3: marked increased signal intensity |
| Bone marrow edema | Absent  Present |
| Deep Infrapatellar bursitis | Absent  Present |

Abbreviations: mm: millimeter; AP: anteroposterior; IFP: infrapatellar fat pad;

**Table S2. Parameters of the magnetic resonance imaging sequences**

| **Sequence** | **3D PD** | **Axial T2** |
| --- | --- | --- |
| Matrix | 384 X 384 | 320 X 320 |
| Scan plane | Sagittal | Axial |
| FOV (cm) | 15.0 | 12.0 |
| Resolution (mm) | 0.4 X 0.4 X 1.0 | 0.375 X0.375 |
| Slice Thickness (mm) | 1.0 | 2.0 |
| Number of Slices | 120 | 32 |
| TE (msec) | 30.0 | 88.0 |
| TR (msec) | 1200.0 | 9378.0 |
| Bandwidth (± kHz) | 83.33 | 81.36 |
| NEX | 0.5 | 1 |
| Fat saturation | Yes | yes |

Abbreviations: PD: Proton density; FOV: field-of-view; TE: echo time; TR: repetition time; NEX: number of excitations

**Table S3. Description of Inter-rater reliability calculations**

| **Category** | **Statistics** |
| --- | --- |
| Binary imaging variables | Cohen’s k values were interpreted using the following cutoffs: <0 poor; 0.01 to 0.20 slight; 0.21 to 0.40 fair; 0.41 to 0.60 moderate; 0.61 to 0.80 substantial; and 0.81 to 1.00 almost perfect or 1.00 perfect [2]. |
| Ordinal imaging variables | Gwet’s AC2 values were interpreted using the following cutoffs: <0 poor; 0.01 to 0.20 slight; 0.21 to 0.40 fair; 0.41 to 0.60 moderate; 0.61 to 0.80 substantial; and 0.81 to 1.00 almost perfect or 1.00 perfect [2]. |
| Continuous imaging variables | Interclass correlation coefficient (ICC) was interpreted using the following cutoffs: < 0.5 indicates poor reliability, ICC between 0.5 and 0.75 indicates moderate reliability, ICC between 0.75 and 0.9 indicates good reliability, and ICC > 0.90 indicates excellent reliability [3]. |

[2] Landis JR, Koch GG. The measurement of observer agreement for categorical data. Biometrics. 1977;33(1):159-174; PMID:843571

[3] Koo TK, Li MY. A Guideline of Selecting and Reporting Intraclass Correlation Coefficients for Reliability Research. J Chiropr Med. 2016;15(2):155-163; PMID:27330520

**Appendix B: results**

**Table S1. Inter-rater reliability (IRR) for imaging findings**

| **Imaging features** | **IRR (95% confidence interval [CI])** |
| --- | --- |
| Tendon AP thickness (mm) | 0.83 (0.73-0.90) |
| The presence of intratendinous calcification | 0.84 (0.69-0.96) |
| The presence of patella erosions | 0.83 (0.69-0.95) |
| The level of Doppler flow | 0.81 (0.75-0.88) |
| The presence of Focal tendon disruption | 0.88 (0.72-1.00) |
| The presence of >50% focal tendon disruption | 0.79 (0.77-1.00) |
| The presence of IFP edema | 0.86 (0.82-0.91) |
| The presence of bone marrow edema | 0.94 (0.85-1.00) |
| The presence of deep Infrapatellar bursitis | 0.89 (0.78-0.97) |

Abbreviation: IFP: infrapatellar fat pad

**Table S2. Estimated mean difference from 24 weeks to baseline using linear mixed-effect models**

| Imaging features | Change in VAS-SLDS | | Change in VISA-P score | |
| --- | --- | --- | --- | --- |
|  | Contrasts within subgroups  Mean diff. (95% CI) | Contrasts between subgroups  Mean diff. (95% CI) | Contrasts within subgroups  Mean diff. (95% CI) | Contrast between subgroups  Mean diff. (95% CI) |
| Tendon AP thickness  **Normal**  **Tendon thickening** | -2.6 (-3.9 to -1.2)  -2.7 (-3.4 to -1.9) | 0.1 (-1.4 to 1.6) | 26.7 (17.7 to 35.7)  22.5 (17.5 to 27.5) | 4.2(-6.1 to 14.5) |
| Intratendinous calcifications  Absent  Present | -2.7 (-3.4 to -1.9)  -2.5 (-3.8 to -1.2) | -0.1 (-1.6 to 1.4) | 25.3 (20.4 to 30.3)  18.0 (9.4 to 26.6) | 7.3 (-2.6 to 17.2) |
| Patellar erosions  Absent  Present | -2.4 (-3.2 to -1.7)  -3.4 (-4.5 to -2.4) | 1.0 (-0.3 to 2.3) | 21.9 (16.7 to 27.2)  26.7 (19.1 to 34.3) | -4.8 (-14.0 to 4.5) |
| Power Doppler flow  No to low (score 0-2)  Moderate to high (score 3-4) | -2.2 (-3.2 to -1.2)  -3.0 (-3.8 to 2.3) | 0.8 (-0.5 to 2.0) | 20.8 (13.5 to 28.2)  24.9 (19.6 to 30.3) | -4.1 (-13.2 to 5.0) |
| Focal tendon disruption  Absent  Present | -1.7 (-3.0 to -0.4)  -2.9 (-3.6 to -2.2) | 1.2 (-0.3 to 2.7) | 23.4 (14.1 to 32.7)  23.4 (18.5 to 28.4) | -0.1 (-10.7 to 10.5) |
| IFP edema  No to low (grade 0-1)  Moderate to high (grade 2-3) | -2.3 (-3.4 to -1.2)  -2.8 (-3.6 to -2.0) | 0.5 (-0.8 to 1.9) | 18.8 (11.4 to 26.2)  25.9 (20.6 to 31.2) | -7.1 (-16.2 to 2.0) |
| Bone marrow edema  Absent  Present | -2.4 (-3.2 to -1.6)  -3.0 (-4.0 to -2.0) | 0.7 (-0.5 to 1.9) | 23.6 (18.0 to 29.2)  23.3 (16.5 to 30.0) | 0.3 (-8.5 to 9.1) |
| Deep Infrapatellar bursitis  Absent  Present | -3.1 (-4.1 to -2.2)  -2.2 (-3.1 to -1.3) | -0.9 (-2.2 to 0.3) | 21.9 (15.5 to 28.3)  24.8 (18.9 to 30.7) | -2.8 (-11.6 to 5.9) |

Abbreviations: AP: anteroposterior; IFP: infrapatellar fat pad;

**Table S3. Results of the linear mixed-effect model with 24-week change in clinical outcomes according to imaging factor at baseline using the complete dataset**

| **Imaging features** | **VAS-SLDS** | | | **VISA-P** | | |
| --- | --- | --- | --- | --- | --- | --- |
|  | **β (95% CI)** | **P value** | **P value for interaction^a^** | **β (95% CI)** | **P value** | **P value for interaction^b^** |
| Focal tendon disruption  Present vs absent | -0.0 (-0.67 to 0.61) | 0.931 | 0.096 | -0.9 (-4.45 to 2.64) | 0.621 | 0.915 |
| IFP edema  Moderate to high vs no to low | -0.1 (-0.45 to 0.58) | 0.803 | 0.455 | -0.1 (-3.15 to 2.98) | 0.956 | 0.076 |
| Bone marrow edema  Present vs absent | 0.3 (-0.20 to 0.78) | 0.253 | 0.526 | -1.4 (-4.30 to 1.43) | 0.333 | 0.736 |
| Deep infrapatellar bursitis  Present vs absent | 0.2 (-0.31 to 0.63) | 0.516 | 0.123 | 0.0 (-2.82 to 2.91) | 0.976 | 0.533 |

^a^ Likelihood ration test for VAS-SLDS, comparing models with and without the interaction term (imaging predictor x time).

^b^ Likelihood ration test for VAS-SLDS, comparing models with and without the interaction term (imaging predictor x time).

Abbreviations: VAS-SLDS: Visual Analog Score after single-leg decline squat; VISA-P: Victorian Institute of Sports Assessment–Patella; IFP: infrapatellar fat pad.

Figure S1


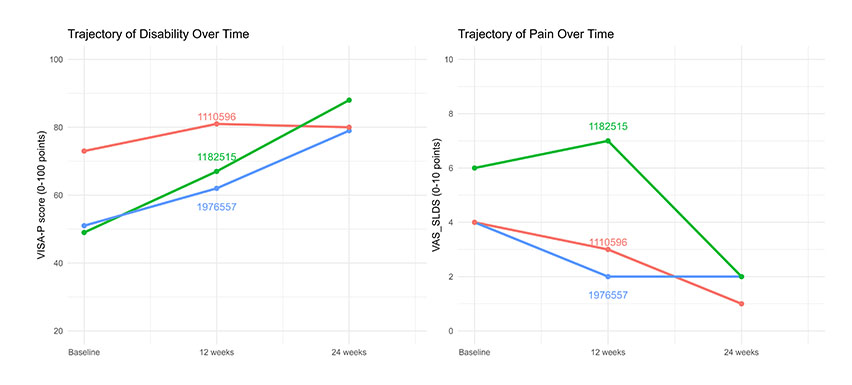


Trajectories of VISA-P and VAS-SLDS scores from baseline to 24 weeks in three athletes with >50% focal tendon disruption at baseline, following exercise therapy. Each line represents an individual athlete’s score trajectory; numbers indicate their study ID. None of the athletes underwent surgery during the 24-week follow-up period. Abbreviations: VAS-SLDS = Visual Analogue Scale after Single-Leg Decline Squat; VISA-P = Victorian Institute of Sport Assessment–Patella.
